# Lynch Syndrome as a Spectrum of Four Distinct Genetic Disorders: Toward Genotype-Guided Precision Management in the NGS Era

**DOI:** 10.3390/cancers18030506

**Published:** 2026-02-03

**Authors:** Yuanyuan Liu, Shengwei Ye, Zhen Liu, Zhen Chen, Xinjun Liang

**Affiliations:** 1 Department of Central Laboratory & Biobank, Hubei Cancer Hospital, Tongji Medical College, Huazhong University of Science and Technology, Wuhan 430079, China; liuyy0710@hotmail.com (Y.L.);; 2 Colorectal Cancer Clinical Research Center of Wuhan, Wuhan 430079, China; 3 Colorectal Cancer Clinical Research Center of Hubei Province, Wuhan 430079, China; 4 Department of Gastrointestinal Surgery, Hubei Cancer Hospital, Tongji Medical College, Huazhong University of Science and Technology, Wuhan 430079, China; 5 Key Laboratory of Pesticide and Chemical Biology of Ministry of Education, School of Life Sciences, Central China Normal University, Wuhan 430079, China; 6 Hubei Key Laboratory of Genetic Regulation and Integrative Biology, School of Life Sciences, Central China Normal University, Wuhan 430079, China; 7 Department of Abdominal Oncology, Hubei Cancer Hospital, Tongji Medical College, Huazhong University of Science and Technology, Wuhan 430079, China

**Keywords:** Lynch syndrome, hereditary cancer syndromes, precision management, genotype–phenotype correlations, risk stratification, genotype-guided surveillance, immunotherapy, mismatch repair deficiency

## Abstract

Background/Objectives: Lynch syndrome (LS), is traditionally managed uniformly despite being caused by pathogenic variants in four distinct mismatch repair (MMR) genes (*MLH1*, *MSH2*, *MSH6*, and *PMS2*). This approach fails to leverage gene-specific characteristics for precision healthcare delivery. This review redefines LS as four distinct genetic syndromes and establishes a genotype-guided precision management framework to optimize risk stratification, surveillance, and therapeutic interventions. Methods: We synthesized molecular, clinical, and outcomes data from the Prospective Lynch Syndrome Database (8500+ carriers; 70,000 person-years), genomic studies characterizing gene-specific mutational patterns, and immunotherapy trials while referencing international guidelines [National Comprehensive Cancer Network (NCCN), European Hereditary Tumour Group (EHTG)/European Society of Coloproctology (ESCP), and European Society for Medical Oncology (ESMO)] to formulate genotype-stratified recommendations. Results: Fundamental molecular differences necessitate differentiated management strategies. *MLH1* deficiency exhibits unique “two-in-one hit” mechanisms driving aggressive tumorigenesis with high interval cancer rates. *MSH2* deficiency presents the highest tumor mutational burden (≈47 mutations per megabase; Mut/Mb) and broadest cancer spectrum. *MSH6* deficiency displays distinctive high-single-nucleotide variant (SNV)/low-insertion–deletion (Indel) patterns often presenting as microsatellite instability-low (MSI-low) or microsatellite stable (MSS), complicating conventional detection. *PMS2* deficiency demonstrates substantial attenuation due to redundancy. These translate into precision interventions: *MLH1/MSH2* carriers require colonoscopy from age 25 at 1–2-year intervals with extended colectomy preferred, while *MSH6/PMS2* carriers can defer surveillance to age 35–40 with longer intervals and undergo segmental resection. Immune checkpoint inhibitors (ICIs) are effective in deficient *MMR* (dMMR)/microsatellite instability-high (MSI-H) tumors across all four MMR genotypes. Conclusions: Genotype-specific precision management optimizes the benefit–burden balance, enhances early cancer detection, reduces overtreatment, and enables personalized genetic counseling, advancing precision healthcare for LS families and addressing critical gaps in hereditary cancer care.

## Simple Summary

Lynch syndrome (LS) is the most common inherited cancer predisposition. Traditionally managed as a single disease, emerging evidence revealed that LS comprises four distinct conditions based on which mismatch repair (MMR) gene is affected. *MLH1* and *MSH2* mutations cause aggressive, rapidly progressing cancers requiring intensive monitoring from age 25, while *MSH6* and *PMS2* mutations lead to slower progression with later onset, allowing for less frequent surveillance starting at age 35–40. This review synthesizes current evidence to propose a genotype-guided framework where each subtype receives tailored management strategies for screening, surgery, immunotherapy, and prevention. Understanding LS as four separate conditions enables personalized care, avoiding unnecessary interventions in lower-risk patients while ensuring adequate protection for high-risk carriers, thereby improving outcomes for patients and families.

## 1. Introduction

Lynch syndrome (LS) is the most common autosomal dominant hereditary cancer syndrome, caused by germline pathogenic variants in four mismatch repair (*MMR*) genes: *MLH1*, *MSH2*, *MSH6*, and *PMS2* [1,2]. *EPCAM* deletions, which epigenetically silence adjacent MSH2, represent a rare alternative mechanism managed clinically as *MSH2* deficiency [3]. Individuals with LS face lifelong elevated risks of colorectal cancer (CRC), endometrial cancer (EC), and various extracolonic malignancies [4,5].

The diagnostic approach for LS has evolved from the Amsterdam criteria and Bethesda guidelines to the current next-generation sequencing (NGS) era [1,2]. NGS enables population-level prospective identification rather than family history-driven inference. Data from the All of Us Research Program suggest a prevalence of approximately 1 in 354, with more than 60% of carriers lacking typical family history [6], fundamentally challenging traditional screening pathways. However, NGS also presents new challenges: variants of uncertain significance (VUSs) have surged beyond current interpretive capacity [7], and “Lynch-like” syndrome (LLS) blurs hereditary–sporadic boundaries [8]. These complexities indicate that tumor phenotype or germline testing alone cannot support individualized management [9,10].

Mounting evidence indicates that while LS is unified by shared mismatch repair (*MMR*) deficiency biology, pathogenic variants in *MLH1*, *MSH2*, *MSH6*, and *PMS2* are associated with meaningful gene-specific differences in carcinogenic pathways, molecular phenotypes, clinical risks, and outcomes [11]. Recent comprehensive reviews have addressed LS management from various perspectives. Notably, Eroğlu et al. provided an excellent multidisciplinary overview organized by organ system and clinical domain [12]. In contrast, our review adopts a fundamentally different genotype-first architecture, positioning the four *MMR* genes as the primary stratification axis. We trace explicit mechanistic links from molecular features to clinical implications, offering not only “what to do” but also “why.” Furthermore, we implement an evidence hierarchy that distinguishes prospective PLSD data from retrospective cohorts and theoretical inferences, enabling clinicians to calibrate confidence in each recommendation.

We believe this framework complements existing reviews and offers a distinct perspective for precision management. Accordingly, we propose viewing LS as a gene-defined spectrum comprising four distinct entities, a conceptual framework intended to guide precision management rather than a strict nosologic redefinition. Based on this framework, we aim to develop genotype-guided recommendations across diagnosis, surveillance, treatment, and prevention to better align clinical intensity with gene-specific risk and biology.

This review synthesizes evidence across a hierarchy of study designs. Level I evidence (randomized controlled trials and meta-analyses) remains limited in LS due to its rarity. Level II evidence (prospective cohort studies), particularly data from the Prospective Lynch Syndrome Database (PLSD), provides the most robust risk estimates and informs most surveillance recommendations herein. Level III evidence (retrospective cohort and case-control studies) supplements areas where prospective data are lacking. Level IV evidence (case series and theoretical inferences from molecular mechanisms) is distinguished clearly when used to generate hypotheses or explain biological rationale. Throughout this review, we explicitly indicate the evidence level supporting each major recommendation to facilitate appropriate clinical translation.

## 2. Diagnostic Innovations and Challenges in the NGS Era

NGS has transformed LS diagnosis from a family history-driven linear workflow into a high-throughput molecular testing model. However, this transformation has produced two major clinical dilemmas—an explosion of VUSs and the challenge of differentiating LLS—while establishing new requirements for test standardization and broad screening implementation.

### 2.1. Historical Enhancement of Diagnostic Capability

The transition from single-gene Sanger sequencing to multigene panel testing has improved testing efficiency and shifted strategies from high-risk population screening to broader population coverage [2,13]. National Comprehensive Cancer Network (NCCN) guideline recommends multigene testing as a preferred initial approach [14].

This transition offers three key advantages: (1) NGS uncovers previously “hidden” carriers lacking typical family histories who would be missed by traditional pathways [13]; (2) multigene panels simultaneously cover all four *MMR* genes (with *EPCAM* deletion analysis typically included), avoiding sequential testing delays and cost; and (3) universal defective *MMR*/microsatellite instability (dMMR/MSI) testing for newly diagnosed CRC and EC markedly increases LS detection rates [15,16].

This subsection draws primarily on retrospective cohort studies and guideline recommendations (Level II–III evidence).

### 2.2. Precision Subtyping of LLS

Accurate identification of true LS carriers and their specific *MMR* gene defect is the premise of genotype-guided precision management. However, 60–70% of patients with dMMR/MSI-H tumors lack identifiable germline pathogenic MMR variants, a condition termed LLS [8,17]. Without resolving LLS etiology, sporadic patients may receive unnecessary lifelong surveillance, while true LS carriers with cryptic variants may be missed.

Two principal molecular causes of LLS have distinct management implications. The first is biallelic somatic inactivation, which represents the predominant cause. Using paired tumor–normal sequencing, Walker et al. assigned definitive molecular subtypes to 86.9% of suspected cases, confirming that most LLS are sporadic cancers with biallelic somatic *MMR* inactivation [8]. Golubicki et al. identified alterations in noncanonical genes (*MSH3* or *POLD1*), underscoring LLS molecular heterogeneity [18]. These patients should be managed as sporadic dMMR cases without intensive LS surveillance or family cascade testing.

The second is cryptic germline events beyond conventional NGS detection, which explain the remaining cases. Long-read sequencing by Te Paske et al. showed that deep intronic variants and structural rearrangements account for approximately 28% of “missing heritability” [9]; recent studies identified an *MSH2* paracentric inversion [19] and an MSH6 retrotransposon insertion [20]. These patients are true LS carriers requiring gene-specific management, as per Section 4.

Constitutional epimutations deserve special attention: among dMMR CRC patients aged ≤55 years with *MLH1* methylation-positive tumors, constitutional *MLH1* promoter epimutation incidence is elevated. Blood-based methylation testing is essential to avoid missed diagnoses [21]. These patients carry heritable risk and require *MLH1*-specific LS protocols.

The integrated use of tumor–germline paired sequencing, long-read sequencing, and methylation testing enables precise etiological attribution and genotype-specific management.

Evidence in this subsection derives from prospective and retrospective cohort studies (Levels II–III); cryptic variant detection relies partly on case series and mechanistic inference (Level IV).

### 2.3. Reclassification Strategies for VUSs

As the NGS scope expands, VUSs in *MMR* genes have risen sharply. ClinVar data (December 2025) indicate VUS proportions ranging from 12.1% (*MSH2*) to 33.6% (*PMS2*), with “conflicting interpretations” across genes. The high *PMS2* proportion likely reflects detection challenges from highly homologous pseudogenes. VUSs misclassification carries bidirectional risk: pathogenic misclassification causes over-surveillance; benign misclassification causes insufficient surveillance.

Classification methods have progressed from the 2015 American College of Medical Genetics and Genomics/Association for Molecular Pathology (ACMG/AMP) general framework to refined systems such as ClinGen–InSiGHT gene-specific standards, enhancing accuracy and consistency [22,23].

As summarized in Table 1, we recommend a four-layer VUSs reclassification framework.

However, challenges remain: lack of unified evidence weighting, high costs and long timelines for functional validation, and scarce data on rare variants [24,27]. Future advances in multicenter data sharing, standardized MAVE rollout, and AI application are expected to improve automation [7,26].

This subsection synthesizes guideline recommendations (Levels II–III) and emerging functional assay data (Levels III–IV); the four-layer framework represents expert consensus informed by available evidence.

### 2.4. Standardization and Integration of Testing Methods

Discordance between immunohistochemistry (IHC) and PCR-based MSI testing reaches 19.3% [31], particularly prominent in MSH6-associated tumors. Due to high single nucleotide variant (SNV)/low insertion deletion (Indel) mutational patterns, such tumors frequently present as MSI-low (MSI-L) or even microsatellite stable (MSS), making conventional PCR assays prone to missed detection [32,33]. In EC, binary “intact/lost” IHC interpretation without detailed reporting of subclonal patterns may miss approximately 21% of LS cases [33].

As summarized in Table 2, we recommended a tiered complementary testing strategy.

AI-assisted technologies offer new possibilities: the DeepPath-MSI deep learning model achieves an AUROC of 0.98, enabling MSI pre-screening [36]. Alouani et al. reported significantly worse immunotherapy efficacy with IHC-MSI discordance, underscoring the necessity of dual testing and TMB/POLE mutation testing in discordant cases [37].

Recommendations derive from retrospective diagnostic accuracy studies (Level III) and emerging AI validation cohorts (Levels III–IV).

### 2.5. Dissemination and Accessibility of Screening

Universal dMMR/MSI screening for newly diagnosed CRC and EC is recommended by major guidelines and increases LS detection rates [2,14,38,39]. However, system-level barriers—variability in pathologist practices, inefficient referral pathways, and inadequate insurance coverage—limit real-world effectiveness; approximately 24% of screening-positive individuals do not receive genetic counseling even under near-ideal conditions [14].

Health economic studies support cost effectiveness. Markov model evaluations indicate that “universal germline screening combined with polygenic risk scores (PGS)” would avert 1.36 CRC cases and 0.65 deaths per 1000 individuals screened, with an incremental cost-effectiveness ratio of $124,415 per quality-adjusted life year and a 69% probability of cost-effectiveness [40].

Key implementation promotes optimizing pathology-to-genetic counseling referral workflows, improving insurance coverage, and developing simplified screening tools suitable for primary care settings.

This subsection draws on health economic modeling studies (Level III) and implementation research (Level III); universal screening recommendations reflect guideline consensus based on accumulated cohort evidence (Levels II–III).

In summary, LS diagnosis in the NGS era requires integrated solutions: (1) tumor–germline comprehensive subtyping to distinguish LS from sporadic LLS; (2) a multilayered VUSs reclassification framework advancing standardization; (3) a tiered testing pathway with IHC as first line and NGS as complementary; and (4) continued enhancement of universal screening accessibility. This diagnostic precision lays the foundation for subsequent genotype-guided management.

## 3. Molecular Heterogeneity of the Four MMR Genes: The Biological Basis of Precision Management

Implementing genotype-guided precision management requires understanding fundamental differences among *MMR* genes in carcinogenic mechanisms and molecular features. Grouping all four genes under a single “Lynch syndrome” concept no longer accurately reflects their diverse biological behaviors.

### 3.1. Diversified Carcinogenic Pathways in LS-Associated CRC

At least three pathways underlie LS-associated CRC [11,41,42,43]: conventional adenomas, with secondary *MMR* inactivation followed by accelerated progression; adenomas initiated with primary *MMR* deficiency; and a rapidly progressive, non-polypoid pathway that bypasses the adenoma stage (especially prominent in *MLH1* carriers). This multi-pathway reality has important implications for understanding surveillance effectiveness and limitations.

At the molecular level, Martin et al. found that sporadic and LS-associated MSI tumors share similar downstream mutational signatures despite different origins; however, LS tumors exhibit reduced interferon-γ-regulated immune pathway activity in the tumor microenvironment (TME) [44]. Epigenetically, Mäki-Nevala et al. demonstrated that methylation changes are already significant in low-grade dysplasia and even MSS adenomas, suggesting epigenetic alterations as early tumorigenesis drivers [45].

### 3.2. MLH1 Deficiency: A Rapid Carcinogenesis Model via a ”Two-in-One Hit” Mechanism

*MLH1*-deficient tumors exhibit a distinctive aggressive carcinogenic strategy. The “two-in-one hit” mechanism proposed by Ahadova et al. exploits chromosomal positional features: *MLH1* and *CTNNB1* occupy proximal loci on chromosome 3p. A single copy number neutral loss of heterozygosity (cnLOH) event can simultaneously cause *MLH1* inactivation and homozygous *CTNNB1* activation, enabling bypass of the conventional adenoma stage for rapid invasive growth [43]. This mechanism explains the higher incidence of interval cancers in *MLH1* carriers.

Key molecular features include the following:

(1) About 50% harbor *CTNNB1* mutations (predominantly codons 41/45), significantly higher than other *MMR* genes [11,42,43,46];
(2) Low APC mutation frequency (11%), contrasting with *MSH2* [46];
(3) *BRAF V600E* is frequent in sporadic *MLH1*-methylated tumors but typically absent in LS–*MLH1* tumors, an important marker distinguishing hereditary from sporadic cases [17,41,47,48].

Evidence derives from tumor genomic profiling studies (Level III) and mechanistic molecular analyses (Level IV).

### 3.3. MSH2 Deficiency: The Molecular Basis of a Broad-Spectrum High-Risk Profile

*MSH2*-deficient tumors show aggressiveness comparable to *MLH1* but with distinct molecular features. Salem et al. reported that tumors with concurrent *MSH2/MSH6* loss have TMB of 46.83 Mut/Mb, approximately double that of *MLH1/PMS2*-deficient tumors (25.03 Mut/Mb), with a correspondingly higher neoantigen load (NAL) [49], providing a molecular basis for immunogenicity and immunotherapy sensitivity.

The driver gene profile is as follows:

(1) *CTNNB1* mutations rare (7%) [46];
(2) *APC* mutations frequent (75%) [46];
(3) Suggests stronger tendency toward conventional adenoma–carcinoma sequence rather than “two-in-one hit”.

Urinary tract susceptibility:

Nikkola et al. found that *TERT* promoter mutations were nearly absent in LS-associated urothelial carcinoma (UC) (5% vs. 83% in sporadic), serving as a discriminative marker; meanwhile, *FGFR3* mutations occur in ~80% of cases, suggesting FGFR inhibitors to be potential targeted therapeutic options [50].

Evidence derives from comparative tumor profiling studies (Level III); therapeutic implications represent hypothesis-generating observations (Level IV).

### 3.4. MSH6 Deficiency: A Distinct Low-Instability Phenotype

*MSH6*-associated tumors exhibit a unique high-SNV/low-Indel mutational pattern [32,42]. This arises from the *MMR* system mechanics: the *MSH2*–*MSH6* heterodimer (MutSα) primarily recognizes base–base mismatches and small insertion–deletion loops, whereas the *MSH2*–*MSH3* (MutSβ) recognizes larger insertions–deletions. When *MSH6* is deficient, MutSα function is lost but MutSβ is retained, predisposing cells to accumulate SNVs rather than the large microsatellite alterations detected by classical PCR-based MSI assays.

Clinical implications include the following:

(1) Often present as MSI-L or even MSS [32,33], explaining the limited MSI-PCR sensitivity;
(2) IHC recommended as first-line screening;
(3) *CTNNB1* alterations significantly less prevalent than in *MLH1* (8% vs. 47%) [42], potentially underlying slower progression and lower interval cancer incidence;
(4) Lower MSI levels and weaker frameshift mutation loads imply fewer frameshift-derived neopeptides, with potential implications for immune checkpoint inhibitors (ICI) responsiveness and vaccine development.

Evidence derives from molecular characterization studies (Level III); immunotherapy implications represent mechanistic inference (Level IV).

### 3.5. PMS2 Deficiency: The Molecular Basis of Attenuated Risk

*PMS2* carriers represent the lowest-risk end of the LS spectrum. This attenuated phenotype has clear molecular bases.

Functional redundancy: *PMS2* forms the MutLα heterodimer with *MLH1* to provide endonuclease activity; however, *MLH3* and *PMS1* can partially substitute for *PMS2*, so the loss of *PMS2* alone has relatively limited impact on genome stability [51,52].

The carcinogenic pattern is as follows:

(1) Tends to follow conventional adenoma–carcinoma sequence;
(2) *PMS2* loss is often a late event rather than an initiating driver;
(3) Low frequency of somatic *KRAS* hotspot mutations supports slow progression [52].

TME features: Immune infiltration is weaker than in *MLH1/MSH2*-deficient tumors, potentially related to lower mutation and NALs, with implications for immunotherapy strategies [52].

Detection caveat: High sequence homology of the *PMS2CL* pseudogene has historically complicated accurate detection, leading to potential underdiagnosis [6].

Evidence derives from functional studies and tumor profiling (Levels III–IV); clinical risk estimates informed by prospective cohort data (Level II).

### 3.6. Genotypic–Molecular Feature Summary

In summary, the four *MMR* genes differ fundamentally in carcinogenic mechanisms, MSI phenotypes, driver mutation profiles, and TME characteristics (Table 3). *MLH1* deficiency drives rapid carcinogenesis via the “two-in-one hit” mechanism; *MSH2* deficiency confers broad-spectrum high risk with elevated TMB/NAL; *MSH6* deficiency produces a distinct low-instability phenotype requiring adapted detection strategies; *PMS2* deficiency shows attenuated risk due to functional redundancy. These molecular differences provide the biological rationale for gene-specific surveillance, treatment, and prevention strategies detailed in subsequent sections.

Overall, this section synthesizes evidence primarily from tumor genomic profiling and molecular mechanistic studies (Levels III–IV), which inform but do not directly establish clinical recommendations.

## 4. Genotype-Guided Precision Clinical Management

**Figure 1:**
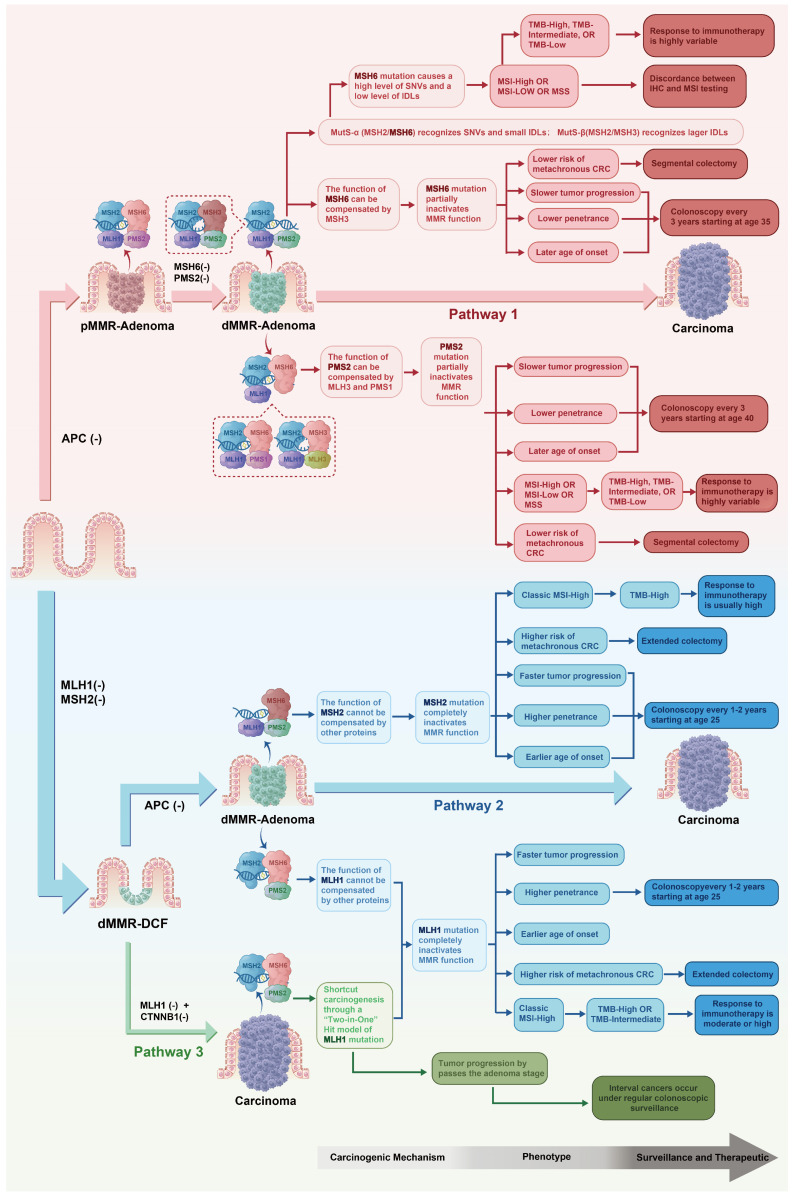
Carcinogenic mechanisms, molecular phenotypes, and clinical management strategies for the four MMR gene defects in LS. Arrows and deepening colors represent the logical flow from molecular mechanisms through clinical phenotypes to management strategies.

The molecular heterogeneity outlined in Section 3 provides the biological rationale for differentiated management. This section describes how to translate these molecular features into precise decisions across risk stratification, surveillance, treatment, and prevention. Figure 1 integrates correspondences among genotype, mechanisms, and management strategies; Table 4 serves as a clinical quick reference for gene-specific management, with detailed epidemiological data, intervention outcomes, and immunotherapy-relevant features presented subsequently.

Figure 1 depicts the correspondence from carcinogenic mechanisms to clinical management for *MLH1*, *MSH2*, *MSH6*, and *PMS2* defects. Pathway 1 (predominantly *MSH6/PMS2*): Owing to functional redundancy, *MMR* is only partially inactivated; *MSH6* exhibits a high-SNV/low-Indel pattern and often shows MSI-L/MSS phenotypes; *PMS2* progresses most slowly, with the lowest penetrance. Both follow the conventional adenoma-carcinoma sequence and thus support segmental resection and less intensive surveillance (beginning at ages 35–40, every 2–3 years). Pathway 2 (predominantly *MSH2*, partly *MLH1*): *MMR* function is completely lost, with rapid progression and high penetrance; tumors present classic MSI-H and the highest TMB, consistent with heightened immunogenicity and robust ICI responsiveness. Surveillance is recommended from age 25, every 1–2 years, with extended colectomy preferred. Pathway 3 (*MLH1*): a unique “two-in-one hit” mechanism–cnLOH on chromosome 3p simultaneously causes *MLH1* inactivation and *CTNNB1* activation–enables direct transformation from the epithelium to carcinomas while bypassing adenomas; this explains interval cancer occurrence and warrants the most intensive surveillance and extended colectomy.

This figure encapsulates the core concept of LS as a spectrum of four hereditary syndromes with distinct molecular features, providing the theoretical framework for genotype-guided precision management.

### 4.1. Risk Stratification: From Population to Individual

#### 4.1.1. Gene-Specific Cancer Risk

Precise risk stratification depends on high-quality prospective cohort data. The Prospective Lynch Syndrome Database (PLSD) is the world’s largest prospective LS cohort, spanning 25 countries with >8500 carriers and >70,000 person-years of follow-up. Table 3 summarizes the PLSD series (2018–2025), providing evidence for genotype-guided risk stratification. From the first 2018 report (3119 carriers) [5] to the 2025 analysis (8438 carriers) [59], this database offers a unique resource for elucidating LS natural history and intervention effects (Table 5).

Important caveat: PLSD-derived risk estimates represent population-level averages and do not fully capture within-genotype heterogeneity. Individual risk may be substantially modified by sex, family history, variant-specific effects, modifier genes, polygenic background, lifestyle, and surveillance adherence. These estimates should serve as a stratification framework rather than deterministic individual predictions.

Practice-shaping insights from PLSD data (Table 6):

First, CRC risk is dichotomized. *MLH1/MSH2* pathogenic variant carriers have cumulative risks by age 75 of 43–57% (high-risk group), whereas *MSH6* (15–20%) and *PMS2* (~10%) are substantially lower (moderate–low-risk group). In a Dutch cohort (*n* = 1117), *MSH6* carriers had extremely low risk at age 40 (men 0.2%; women 0.9%), and meta-analyses confirmed that *MSH6/PMS2* at age 40 is <2% vs. >4% for *MLH1/MSH2* [57,62].

Second, age at onset differs markedly. *MLH1/MSH2* carriers have median diagnostic ages of 48–56 years, whereas *MSH6/PMS2* carriers are delayed to 57–71 years, supporting later surveillance initiation. A Japanese study showed actual diagnostic age is 16.4 years later than NCCN recommendations [63].

Third, the tumor spectrum is gene specific: *MSH2* carriers have elevated risks of urinary tract cancers (18%), and *MSH2* variants account for the highest proportion in central nervous system (CNS) tumors (42.6%); women with *MSH6* have an EC risk of 41–46%; *MLH1* carriers have a standardized incidence ratio (SIR) for pancreatobiliary cancers 46.8 times that of the general population [56]; *PMS2* variants constitute 43.4% of “LS-unrelated tumors,” most of which are MSS with low TMB [64].

Fourth, non-CRC mortality is not negligible: PLSD data show that CRC accounts for only 36% of LS cancer deaths; 10-year survival for pancreatobiliary and brain tumors is only 15–42% [55]. LS management should not overfocus on CRC.

Risk estimates in this subsection derive primarily from prospective cohort data (Level II evidence), representing the highest-quality evidence available for LS risk stratification.

#### 4.1.2. Multifactorial Risk Models

A single-gene genotype is the foundation of risk stratification but is insufficient to precisely predict individual outcomes. Substantial phenotypic heterogeneity persists among carriers with the same genotype, indicating the need to integrate multidimensional information to construct individualized risk profiles. Currently, gene-specific penetrance estimates (e.g., PLSD-derived data) and family history assessment are actionable and integrated into standard genetic counseling; the additional tools discussed below remain investigational and require prospective validation before clinical implementation.

PGS are a promising complementary tool. Helfand et al. demonstrated that PGS significantly improve the identification of individuals at high risk for CRC and perform across diverse ancestries [65]. Lifestyle factors are likewise important; specific dietary patterns and obesity—particularly abdominal adiposity—increase CRC risk in LS, with stronger effects observed in men and *MLH1* carriers [66]. The gut microbiome is an emerging dimension: pks-positive (colibactin-producing) Escherichia coli and other pathobionts are associated with risks of metachronous CRC and adenomas [67,68,69]. Circulating biomarkers also show promise: Kärkkäinen et al. integrated circulating microRNAs (c-miRs) and metabolomics data to construct a predictive model with a concordance index (C-index) of 0.76 [70].

The future direction is to develop dynamic risk models integrating germline genetics, PGS, epigenetics, lifestyle, the microbiome, and circulating biomarkers, enabling continuously updated individualized risk profiles.

Multifactorial risk models currently represent hypothesis-generating research (Levels III–IV evidence); clinical implementation requires prospective validation.

### 4.2. Surveillance Strategy Individualization

#### 4.2.1. Genotype-Stratified Colonoscopy Surveillance

Based on risk stratification, international guidelines (e.g., NCCN; EHTG/ESCP) have established a gene-specific consensus for colonoscopy surveillance [39,58]: *MLH1/MSH2* carriers are recommended to begin at age 25, every 1–2 years; *MSH6* carriers can defer to age 35, every 2–3 years; and *PMS2* carriers can further defer to ages 35–40, every 3–5 years. However, genotype alone is insufficient for surveillance decision making; individualized modifiers—including family history of early-onset cancer, prior adenomas (especially advanced lesions), variant-specific penetrance, and realistic adherence patterns—should be integrated into personalized recommendations and may warrant earlier or more intensive surveillance even in *MSH6/PMS2* carriers. Overall clinical evidence supports genotype-stratified surveillance as a population-level framework: Goverde et al. reported no CRC among *MSH6/PMS2* carriers during 6 years of follow-up [71], whereas Liu et al. found that 16% of *MSH6/PMS2*-related CRCs were diagnosed before age 35 [72], indicating that residual early-onset risk exists and individualized assessment remains indispensable.

PLSD analyses found no significant association between surveillance interval and CRC stage (*p* = 0.34) [61]. However, the lack of statistical significance should not be interpreted as evidence that different surveillance intervals are clinically equivalent. The limited number of CRC cases (*n* = 218) and the absence of genotype-stratified analyses may have reduced power to detect moderate interval–stage associations. Moreover, carcinogenic pathways and velocity differ across *MMR* genotypes; for example, *MLH1*-associated CRC may arise rapidly via the “two-in-one hit” mechanism and bypass a detectable adenoma phase, potentially altering interval–outcome relationships. In addition to interval length, surveillance benefit depends on procedural quality, lesion detection rates, and adherence. Future studies incorporating genotype-stratified designs and standardized quality metrics are needed to inform optimal personalized surveillance intervals.

Table 7 summarizes the impact of intervention strategies on clinical outcomes.

These findings reinforce the rationale for genotype-guided surveillance intensity and surgical decision making within LS precision management frameworks.

Surveillance recommendations derive from guideline consensus informed by prospective cohort data (Level II evidence); interval–outcome analyses remain limited by sample size and study design (Level III).

#### 4.2.2. Emerging Surveillance Technologies (Investigational)

Multiple emerging technologies are being developed to address the limitations of conventional colonoscopy; however, these approaches remain investigational and are not yet validated for routine clinical use. In liquid biopsy, Boeri et al. reported that plasma-based MSI testing achieved a negative predictive value (NPV) of 93%, aiding identification of low-risk patients and potentially enabling safe extension of endoscopic intervals [73]. Regarding fecal biomarkers, analysis of volatile organic compounds (VOCs) shows high sensitivity and NPV for advanced neoplasia [74]. In addition, AI-assisted endoscopy (computer-aided detection) can improve recognition of hard-to-detect lesions, such as serrated lesions, including sessile serrated lesions (SSLs) [75]. Collectively, these tools may support risk-adjusted surveillance intervals tailored to genotype and individual risk profiles.

Emerging technologies represent early-phase validation studies (Level III–IV evidence); integration into clinical practice requires prospective trials.

#### 4.2.3. Surveillance of Extracolonic Cancers

Extracolonic cancer surveillance should adopt a genotype-stratified strategy, though current recommendations derive largely from retrospective cohorts and expert consensus rather than prospective screening trials; some organ-specific approaches remain investigational. Urinary tract (emphasis on *MSH2*): Hall et al. showed that urinary MSI analysis achieved 100% sensitivity for asymptomatic UC, outperforming traditional microhematuria testing at 25% [76]; bilateral upper tract urothelial carcinoma (UTUC) is significantly more frequent in LS than in sporadic cases (23% vs. 8%) and should serve as an LS screening cue [77]. Gynecologic (emphasis on *MSH6*): Kauppinen et al. found that 47% of patients harbored cancer-associated variants detectable in histologically normal endometrium, supporting the value of precancer molecular surveillance [78]. Nervous system: The prevalence of dMMR is 2.6% among glioblastoma (GBM) patients younger than 50 years and rises to 12% in those younger than 40 years; screening for dMMR is recommended in young GBM patients [79].

Extracolonic surveillance recommendations derive from retrospective cohort and case-control studies (Level III evidence); prospective validation of screening efficacy is ongoing.

### 4.3. Precision in Surgical Decision Making

Surgical extent selection for first-episode CRC in LS is shifting from a one-size-fits-all model to genotype-stratified precision decision making based on substantial differences in metachronous CRC risk across genotypes. Recommendations for *MLH1/MSH2* carriers are supported by prospective cohort data and guideline consensus, whereas data for *MSH6* and neoadjuvant ICI strategies remain hypothesis generating and require prospective validation.

For high-risk genotypes (*MLH1/MSH2*), the latest PLSD data show that extended colectomy significantly reduces metachronous CRC risk [59]: in *MLH1* carriers, risk decreases from 69% to 25% (absolute reduction 44%); in *MSH2* carriers, it decreases from 65% to 15% (absolute reduction 51%). Mainstream guidelines (ESMO and EHTG/ESCP) recommend prioritizing extended colectomy for these patients [38,39].

For moderate–low-risk genotypes (*MSH6/PMS2*), traditional practice supports standard segmental colectomy. In the Icelandic cohort, 97% of *MSH6/PMS2* patients underwent segmental colectomy, with very low metachronous cancer risk [60]. However, PLSD data show that among *MSH6* carriers, metachronous CRC risk was 0% in the extended colectomy group versus 32% in the segmental colectomy group (*p* = 0.051) [59]. Given borderline significance and limited numbers, this finding is hypothesis generating and requires prospective validation before informing clinical guidelines.

Surgical planning should therefore emphasize shared decision making, weighing potential risk reduction against quality-of-life outcomes (bowel function, lifestyle impact, and patient preferences). If high-quality colonoscopic surveillance and strong adherence are achievable, segmental colectomy is a reasonable option [80]; multicenter retrospective data demonstrate that neoadjuvant ICI therapy can achieve complete clinical response in over 50% of dMMR rectal cancers (including LS patients), supporting the feasibility of organ preservation and nonoperative management [81]; younger patients should also balance the long-term function outcomes after extended colectomy against potential reductions in metachronous CRC risk. Overall, decisions should integrate genotype-specific risk, surveillance feasibility/adherence, patient preferences, and evolving neoadjuvant options rather than fixed genotype-based algorithms.

Surgical recommendations for *MLH1/MSH2* derive from prospective cohort data (Level II evidence) and guideline consensus. Recommendations for *MSH6/PMS2* are based on retrospective cohorts with limited sample sizes (Level III evidence); the potential benefit of extended colectomy in MSH6 requires prospective validation. Neoadjuvant ICI data derive from multicenter retrospective series (Level III).

### 4.4. Immunotherapy Application and Optimization

#### 4.4.1. Efficacy of ICIs in LS-Associated Tumors

ICIs have produced a transformative advance in treating LS-associated dMMR/MSI-H tumors. As an FDA-approved pan-tumor biomarker, dMMR/MSI-H guides ICI selection. In LS-associated CRC, PD-1 inhibitors achieve an objective response rate (ORR) of 70.7%, and neoadjuvant therapy yields 3-year overall survival (OS) and progression-free survival (PFS) rates of 100% [82]. Efficacy is driven by the dMMR phenotype and is independent of whether the defect is germline or somatic in origin [83].

Nevertheless, not all dMMR tumors respond to ICIs [84]. The BRAF V600E mutation is a clear adverse prognostic factor: median overall survival (mOS) is markedly lower in carriers than non-carriers (19 months vs. 113 months) [83]. Mucinous differentiation or signet-ring cell histology is associated with a lower ORR, indicating that histologic features may influence efficacy [82]. In a multicenter retrospective analysis, Lemaire et al. reported a pathological complete response (pCR) rate of 42% for neoadjuvant ICIs in non-metastatic dMMR colon cancer—lower than rates reported in clinical trials—and found mucinous components to be significantly associated with resistance [85].

Even when the ICI is successful, LS patients remain at risk for subsequent malignancies. Prospective studies show that 8–12% of patients develop new malignancies during or after ICIs, 39% have precancerous polyps, and prior versus subsequent tumors rarely share FSDNs, with 98.8% non-overlap [86,87]. Cause-of-death analyses indicate that 43% of deaths are due to pMMR (proficient MMR)/MSS cancers and 46% to dMMR cancers (of which 70% are ICI resistant) [88], underscoring that pMMR tumors are an underestimated cause of mortality and that LS management should not focus solely on dMMR tumors. These observations motivate exploration of genotype-informed strategies to optimize ICI use.

ICI efficacy data derive from prospective trials and multicenter retrospective analyses (Level II–III evidence). Resistance factor associations are based on retrospective cohorts (Level III).

#### 4.4.2. Genotype-Specific ICI Considerations (Hypothesis Generating)

Emerging evidence suggests systematic differences in molecular features among tumors with distinct *MMR* gene defects (Table 8) that could theoretically influence ICI responses; however, these observations are hypothesis generating, and no prospective trials have directly compared ICI efficacies across *MLH1*, *MSH2*, *MSH6*, and *PMS2* genotypes. Importantly, all patients with dMMR/MSI-H tumors remain candidates for ICI therapy based on tumor phenotype, irrespective of germline genotype; current evidence does not support withholding or modifying ICIs based on which *MMR* gene is affected.

Salem et al. reported that tumors with concurrent *MSH2/MSH6* deficiency have a TMB of approximately 47 Mut/Mb—nearly double that of *MLH1/PMS2*-deficient tumors (25 Mut/Mb)—with correspondingly higher neoantigen load (NAL) [49]; given that TMB and NAL are established predictors of ICI response, *MSH2*-associated tumors may theoretically have the highest sensitivity, although this remains unvalidated in LS-specific cohorts. *MSH6*-associated CRC exhibits a distinctive of high-SNV/low-Indel mutational pattern [32,42]; theoretically, low Indel burden could reduce frameshift-derived neopeptides (FSDNs), a major source of immunogenic neoantigens in dMMR tumors. However, this inference rests on unproven assumptions—that FSDNs are the primary drivers of ICI response and that quantitative differences translate into clinical efficacy differences—and SNV-derived neoantigens may partially compensate; until prospective data demonstrate actual efficacy differences, *MSH6*-associated dMMR tumors should be treated equivalently to other dMMR tumors. Limited data suggest that *PMS2*-associated tumors may exhibit weaker immune infiltration and a higher proportion of “immune desert” phenotypes [52,90]; if confirmed, such tumors might benefit from combination immunomodulatory strategies, although this remains a conceptual consideration requiring prospective evaluation.

Genotype-specific ICI efficacy hypotheses derive from molecular profiling studies (Level III) and theoretical inference (Level IV). Prospective validation is essential before clinical application.

#### 4.4.3. ICIs in LS-Associated Rare Cancers (Limited Evidence)

The effectiveness of ICIs has extended beyond CRC and EC to multiple rare cancers, though evidence in these settings is based primarily on case series and small retrospective cohorts and should be considered hypothesis generating. In breast cancer, approximately 38.5% of LS-associated cases are driven by dMMR; even hormone receptor-positive, programmed death-ligand 1 (PD-L1)-negative tumors with an immunologically cold phenotype may benefit if multi-omic testing confirms dMMR/MSI-H [91]. In sarcomas, LS-associated undifferentiated pleomorphic sarcoma (UPS) has demonstrated an ORR of 50% to ICIs [92]. In nervous system tumors, primary dMMR in pediatric and adolescent high-grade gliomas is largely attributable to LS, and immunotherapy has been associated with improved survival [93]. In UC, pancreatic cancer, and lung cancer, dMMR-defined subsets constitute established indications for ICIs [94,95,96].

Rare cancer ICI data derive primarily from case series and small retrospective cohorts (Level III–IV evidence); larger validation studies are needed.

### 4.5. Prevention Strategies

#### 4.5.1. Chemoprevention

Aspirin is currently the only LS chemopreventive agent supported by high-level evidence. The CAPP2 trial demonstrated that taking 600 mg of aspirin daily for at least 2 years can reduce long-term CRC risk by approximately 60% [41]. However, real-world adoption is suboptimal: only about 25.7% of LS patients take aspirin regularly, and dosing is often suboptimal [97]. Aspirin uptake appears more influenced by patients’ subjective perceptions than by objective genetic risk, underscoring the importance of health education and clinician–patient communication.

Optimization strategies include the following:

Dose: the CAPP3 trial is evaluating whether lower doses (100 mg or 300 mg) can maintain efficacy while reducing adverse effects.

Population selection: it is important to prioritize high-risk genotypes (*MLH1/MSH2*) given their elevated CRC risk.

Adherence: practitioners should implement digital reminders and regular follow-up interventions to sustain long-term use.

Aspirin chemoprevention is supported by a randomized controlled trial (Level I evidence). Dose optimization and genotype-specific efficacy data are pending from ongoing trials.

#### 4.5.2. Immunoprevention (Investigational)

Preventive vaccines based on shared FSDNs represent a promising direction in LS immunoprevention; however, current evidence remains proof of concept and is not yet sufficient for routine clinical use. Recurrently mutated coding microsatellite loci in MSI tumors provide a rationale for broadly applicable vaccines, while individualized optimization may require integration of human leukocyte antigen (HLA) typing, immune escape mechanisms, and nonsense-mediated decay (NMD) regulation [98]. Key uncertainties for clinical translation include long-term immune safety and durability (e.g., persistence of immune memory, need for booster dosing, and risk of immune-related adverse events such as autoimmunity), which require extended follow-up in prevention trials. The biological rationale is supported by evidence that shared neoantigens can arise in precancerous lesions [99]; LS carrier mucosa shows an “immune surveillance” state enriched for cytotoxic mucosal-associated invariant T (MAIT) cells associated with delayed tumorigenesis [100,101]; and systematic screening has identified immunogenic neoantigen libraries covering common HLA alleles [102]. Early-phase studies such as the NOUS-209 vaccine program highlight feasibility; De Marco et al. reported that targeted frameshift mutations are shared across CRC and UC, supporting potential expansion beyond CRC [103]. Although immunogenic frameshift targets may be lost in metachronous tumors due to immunoediting, subsequent tumors often acquire new targetable frameshift events, supporting the concept of preventing metachronous malignancies while acknowledging the need for prospective validation.

Immunoprevention data derive from preclinical studies and early-phase trials (Level III–IV evidence). Prospective validation of clinical efficacy is required.

#### 4.5.3. Genotype-Specific Prevention Strategies (Hypothesis Generating)

Preventive strategy design should account for genotype differences, though the considerations discussed below are theoretical and require prospective validation before clinical application. MSH6 deficiency yields fewer frameshift mutations (a high-SNV/low-Indel pattern), which may attenuate the efficacy of FSDN vaccines; future development may therefore require individualized vaccines targeting SNV-derived neoantigens. Genotype-specific features of the TME may also influence vaccine effectiveness; immune infiltration is weaker in PMS2-associated tumors, and combining vaccination with immunomodulatory agents may be necessary to enhance responses.

The ultimate goal is to build a combination-based precision prevention system—aspirin plus vaccines plus lifestyle interventions—integrated with dynamic monitoring technologies such as liquid biopsy (e.g., circulating tumor DNA [ctDNA], and VOCs) to achieve effective immune interception in high-risk individuals.

Genotype-specific prevention considerations represent theoretical inference (Level IV evidence) requiring prospective validation.

In summary, clinical management of LS is shifting from a one-size-fits-all model to tailored, genotype-guided care. High-risk genotypes (MLH1/MSH2) require earlier, more intensive surveillance and more aggressive surgery; moderate–low-risk genotypes (MSH6/PMS2) can appropriately reduce surveillance intensity, prioritizing organ- and function-preserving approaches. Immunotherapy offers new hope for dMMR tumors, but genotype-specific differences in efficacy require clarification through prospective studies. The integration of chemoprevention and immunoprevention may enable a transition from treating established disease to preventing disease. Although a genotype-guided framework has been preliminarily established, key knowledge gaps remain on the path to true individualized medicine and will be discussed in Section 5.

Overall, Section 4 recommendations are supported by varying evidence levels: risk stratification and aspirin chemoprevention by Level I–II evidence; surveillance and surgical strategies by Level II–III evidence; and immunotherapy genotype differences and immunoprevention by Level III–IV evidence requiring prospective validation.

## 5. Current Challenges and Future Directions

Despite significant advances in LS precision management, critical knowledge gaps remain on the path to truly individualized care. At the therapeutic level, genotype-specific differences in sensitivity to ICI are largely hypothesis generating and currently represent the most significant gap impeding a comprehensive genotype-guided framework.

### 5.1. Key Knowledge Gaps

First, high-quality evidence is lacking for the immunotherapy sensitivity of MSH6- and PMS2-associated tumors. The high-SNV/low-Indel mutational pattern of MSH6 implies a reduced load of FSDNs, and immune infiltration appears weaker in PMS2-associated tumors, both of which could theoretically influence ICI and vaccine efficacy. Current evidence is predominantly retrospective, with few targeted prospective studies. As the proportion of carriers with these genotypes increases, the clinical impact of this gap is growing.

Second, risk prediction models have yet to achieve multidimensional integration. Existing assessments primarily rely on single-genotype stratification and cannot precisely predict individual outcomes. PGS, epigenetic modifications, lifestyle factors, and the microbiome may all modulate risk, yet validated models that integrate these dimensions are lacking. There is a need to develop continuously updated, comprehensive predictive tools in large, multi-ancestry cohorts.

Third, standardization and throughput for functional validation of VUSs remain bottlenecks. Current in vitro assays are largely low throughput and insufficient to meet the scale of VUSs reclassification; methodological and standard differences across laboratories limit comparability; and reclassification depends heavily on cumulative evidence and multicenter data sharing. High-throughput functional genomics and AI-driven variant effect prediction are promising, but translation from development to clinical validation will take time.

Fourth, the long-term safety and efficacy of immunopreventive strategies remain to be established. Preventive vaccines based on FSDNs show considerable promise, and early clinical trials (e.g., NOUS-209) are underway; however, key questions—including long-term protective efficacy, optimal vaccination timing, combination with chemoprevention, and genotype-specific effectiveness—require large prospective trials. Whether vaccine-induced immune responses precipitate autoimmune adverse events also requires sustained safety monitoring.

Finally, accessibility and equity in precision management must not be overlooked. Access to advanced testing and individualized care varies substantially across health systems; approximately 24% of screening-positive individuals do not receive genetic counseling [14]. Global dissemination of LS precision management will require policy support to reduce these disparities.

### 5.2. Priority Research Directions

To address the above knowledge gaps, the following research directions should be prioritized.

At the clinical trial level, genotype-specific immunotherapy trials targeting MSH6/PMS2 carriers are needed; Phase II/III prevention trials of frameshift-derived neopeptide (FSDN) vaccines should incorporate genotype-stratified analyses; moreover, trials such as CAPP3 will provide key evidence to optimize aspirin-based prevention. Prior to the availability of direct prospective evidence, it is not recommended to adjust ICI treatment decisions solely on the basis of genotype.

Genotype-specific natural history studies based on large prospective cohorts (such as the ongoing expansion of PLSD) are needed to further quantify organ-specific risks by genotype; the development of dynamically updated risk prediction models integrating multi-omics data should be initiated, aiming to achieve truly individualized risk profiling in clinical practice; meanwhile, international standardization and high-throughput platform construction for functional validation of VUSs should be prioritized as cross-institutional collaborations.

At the basic and translational research level, these efforts should be paralleled to ensure that cohort-derived insights, dynamic multi-omics risk modeling, and standardized high-throughput VUSs functional validation are methodologically robust and clinically translatable.

### 5.3. Clinical Translation Pathways

Translating research findings into clinical practice requires a systematic implementation pathway: at the diagnostic level, prioritize a tiered, IHC-first, NGS-complementary workflow; at the management level, embed genotype-specific surveillance reminders into electronic health record (EHR) systems; and at the service level, establish an integrated, closed-loop pathway connecting genetic counseling, multidisciplinary team (MDT) consultation, and long-term follow-up. Genotype-guided LS management should be implemented as an iterative framework that is continuously refined as prospective genotype-stratified trials, expanded cohort evidence, and emerging biomarkers mature rather than as a fixed algorithm. Collectively, these steps operationalize precision management and support its evolution from an academic concept to sustainable routine care.

## 6. Conclusions

Driven by NGS, the diagnosis and management of LS are shifting from one-size-fits-all approaches to genotype-guided precision care. While LS remains unified by shared mismatch repair (MMR) deficiency biology, accumulating evidence supports clinically meaningful gene-specific differences across MLH1, MSH2, MSH6, and PMS2.

In this framework, MLH1 is linked to rapid carcinogenesis via a “two-in-one hit” mechanism; MSH2 to a broad-spectrum, high-risk profile; MSH6 to a high-SNV/low-Indel profile often presenting with MSI-L or MSS; and PMS2 to attenuated penetrance with later onset, which provides a rationale for differentiated management.

In the NGS era, LS care is increasingly complicated by the surge of variants of uncertain significance (VUSs) and by “Lynch-like” dMMR/MSI-H tumors without a detectable germline cause (LLS), requiring integrated tumor–germline testing and standardized variant interpretation/reclassification to distinguish hereditary from sporadic cases and avoid over- or under-management. With this diagnostic foundation, genotype-guided management can be applied across the care continuum: surveillance (risk-stratified colonoscopy already incorporated into international guidelines), treatment (ICIs for dMMR/MSI-H tumors), and prevention (aspirin chemoprevention and FSDN-based vaccines as complementary strategies, although immunoprevention remains investigational). Finally, genotype-guided precision management in LS has value beyond this syndrome by offering an implementation template that could be adapted to other hereditary cancer syndromes. The comparison with hereditary breast and ovarian cancer (HBOC) is conceptual rather than a call for guideline extrapolation: as LS differs by the affected MMR gene, HBOC also differs meaningfully by BRCA1 versus BRCA2 in ways that extend beyond cumulative risk to tumor biology and treatment vulnerabilities. More broadly, this perspective supports a transferable principle—matching surveillance and interventions to gene-specific penetrance and biology—while emphasizing that each syndrome still requires its own evidence base and independently developed recommendations. Conceptually, biomarker-guided therapy illustrates the same logic across syndromes (e.g., dMMR/MSI-H for immunotherapy in LS and HRD for PARP inhibitors in HBOC). Future research should test how germline–somatic integration can further refine genotype-guided prevention and treatment within syndrome-specific evidentiary standards.

## Figures and Tables

**Table 1:** VUSs reclassification framework.

Layer	Name	Methods/Tools	Technical Details	Key Outcomes	Ref.
1	Theoretical prediction	SIFT, PolyPhen-2, Mutation Assessor, and PROVEAN	Computational algorithms for initial screening	Multi-algorithm complementary use advocated	[7]
2	Functional validation	CIMRA	Cell-free in vitro MMR activity assay	Activity < 25% = pathogenic; 88% MSH6 classification	[24]
		inCAMA	CRISPR-edited variants in hESCs	Direct pathogenicity probability scores	[25]
		MAVE	Saturation mutagenesis for MSH2	74% VUSs definitively classified	[26]
		Tumor profiling	MSI/MMR-IHC/WES signature analysis	Identifies MMR deficiency and second hits	[27]
3	Epidemiologic cross-check	Population AF thresholds	Gene-specific thresholds (PM2, BS1, and BA1)	BS1 contributes most to benign reclassification	[23,28,29]
4	Integrative decision making	Bayesian framework	Multifactor likelihood analysis	Quantitative probability; example: MLH1 in-frame deletion reclassified VUSs → pathogenic via AlphaFold + functional + family analysis	[24,28,30]

**Table 2:** Tiered complementary testing strategy for MMR deficiency detection.

Tier	Purpose	Methods	Key Criteria	Outcomes/Notes	Ref.
1st	Screening	IHC	MLH1, MSH2, MSH6, and PMS2 expression	Prioritized initial screening	—
2nd	Confirmation	NGS-based MSI + TMB	MSI borderline: 8.7–13.8%; TMB: ≥10 Mut/Mb	Accuracy: 92% → 99.6% with TMB	[34]
3rd	Etiologic clarification	Tumor–germline subtyping	Comprehensive analysis	For unresolved cases	—
Auxiliary enhancement method	CNV detection	MLPA	Exon-level CNV	Identifies ~41% additional pathogenic variants; compensates NGS limitations	[35]

**Table 3:** Gene-specific molecular and diagnostic characteristics of LS.

Feature	*MLH1*	*MSH2*	*MSH6*	*PMS2*	Refs.
CARCINOGENIC MECHANISM					
Primary pathway	“Two-in-one hit”: 3p cn-LOH → simultaneous *MLH1* loss + *CTNNB1* activation	Adenoma pathway with early MMR loss; rapid progression	Adenoma pathway; MMR loss may be late event	Classic adenoma–carcinoma sequence	[42,43,46,51,52]
Can bypass adenoma stage	Yes (common)	Rare	Rare	Rare	[43]
MSI PHENOTYPE and DETECTION					
Typical MSI-PCR result	MSI-H	MSI-H	MSI-L or MSS	MSI-H	[32,33,41]
IHC-MSI discordance	Low	Low	High	NR	[31,33]
Recommended detection	Standard IHC/MSI	Standard IHC/MSI	IHC preferred; NGS-MSI if IHC equivocal	Standard IHC/MSI; beware pseudogene	[32,34,52]
DRIVER MUTATION PROFILE					
*CTNNB1* frequency	~50%	~7%	~8%	NR	[11,42,43,46]
*APC* frequency	Low	High	NR	NR	[11,43]
DIAGNOSTIC DIFFERENTIATION					
LS vs. sporadic markers	Sporadic: *BRAF* V600E + *MLH1* methylation; LS: *BRAF* absent	NR	NR	NR	[17,41,47,48]
Organ-specific markers	NR	UC: TERT promoter absent, *FGFR3* ~80%	NR	NR	[50]
Testing caveats	Check *MLH1* methylation in all MLH1-deficient tumors	NR	High IHC-MSI discordance; may miss with PCR-MSI alone	PMS2CL pseudogene interference	[20,32,52]

Table note: IHC, immunohistochemistry; MSI, microsatellite instability; LS, Lynch syndrome; UC, urothelial carcinoma; NR, not reported.

**Table 4:** Summary of gene-specific management strategies (clinical quick reference).

Dimension	*MLH1*	*MSH2*	*MSH6*	*PMS2*
CRC risk level	High (75 years: 46–57%) [5,53]	High (75 years: 43–51%) [5,53]	Moderate (75 years: 15–20%) [4,5]	Low (80 years ∼10%) [54]
Age at onset	Early (median 47–55 years) [5,55]	Early (median 54–56 years) [5,55]	Late (median 55–61 years) [4,55]	Late (median 66–71 years) [54,55]
Tumor spectrum predilection	Upper gastrointestinal and biliary–pancreatic [5,56]	Genitourinary and prostate [5,50,55]	Endometrial [4,55,57]	Predominantly limited to CRC/EC [52,54]
Colonoscopy initiation	25 years [39,58]	25 years [39,58]	35 years [39,58]	35–40 years [39,58]
Colonoscopy interval	1–2 years [39,58]	1–2 years [39,58]	2–3 years [39,58]	3–5 years [39,58]
Initial CRC surgical approach	Extended colectomy [38,59]	Extended colectomy [38,59]	Segmental resection (individualized risk–benefit consideration) [59]	Segmental resection [59,60]
TMB	High [49]	Highest [49]	NR	NR

Table note: CRC, colorectal cancer; TMB, tumor mutation burden; CRC risk and age-at-onset data are primarily from PLSD series [4,5,53,55]; surveillance strategies derive from EHTG/ESCP guidelines [39]; surgical strategies based on PLSD 2025 [59] and ESMO guidelines [38]; TMB derives from reference [49]. NR, not reported.

**Table 5:** Characteristics of studies included in the PLSD analysis (2018–2025).

Study	Journal	Year	Carriers (*n*)	Person-Years	Mean Follow-Up (Years)	Key Contribution
Møller et al. [5]	Gut	2018	3119	24,475	7.8	First large-scale gene-specific risk stratification
Seppälä et al. [61]	Hered Cancer Clin Pract	2019	6350	51,646	NR	Association between surveillance interval and stage at diagnosis
Dominguez-Valentin et al. [4]	Genet Med	2020	6350	51,646	NR	Integration of multicenter risk data across 18 countries
Møller et al. [53]	Hered Cancer Clin Pract	2022	8153	67,604	8.3	Updated cumulative risk estimates
PLSD Collaborative [59]	BJS	2025	8438	65,370	7.8	Surgical strategy and metachronous cancer risk
Dominguez-Valentin et al. [55]	eClinicalMedicine	2023	8500	71,713	8.4	Mortality and survival analysis

Table note: Later studies included carriers from earlier cohorts and extended the follow-up duration.

**Table 6:** Gene-specific cancer risks in LS: cumulative incidence and age at diagnosis from PLSD.

Panel A: Gene-Specific Risks for Colorectal and Endometrial Cancer in LS						
Cancer Type	Risk Parameter	*MLH1*	*MSH2*	*MSH6*	*PMS2*	Clinical Implication
Colorectal	Cumulative risk by age 75	46–57% [5,53]	43–51% [5,53]	15–20% [4,5]	∼10% [54]	Core basis for risk stratification
Colorectal	Median age at diagnosis	48–55 years [5,55]	54–56 years [5,55]	57–61 years [4,55]	66–71 years [54,55]	Basis for surveillance initiation age
Colorectal	Risk before age 50	Significant [5]	Significant [5]	Lower [4]	Very low [54]	Early surveillance required for *MLH1/MSH2*
Endometrial	Cumulative risk by age 75	37–43% [5]	49–57% [5,55]	41–46% [4,55]	13–26% [54]	High risk for women with *MSH2/MSH6*
Endometrial	Median age at diagnosis	52 years [5]	52 years [5]	60 years [4]	61 years [54]	Later onset in *MSH6*
**Panel B: Gene-specific risks for extracolonic cancers in LS**						
**Cancer Type**	**Risk Parameter**	***MLH1***	***MSH2***	***MSH6***	***PMS2***	**Clinical Implication**
Ovarian	Cumulative risk by age 75	10–11% [5]	17% [5,55]	11–13% [4]	0–3% [54]	Highest in *MSH2*
Urinary tract	Cumulative risk by age 75	5% [5]	18% [55]	3–8% [4]	0% [54]	Marked predilection in *MSH2*
Gastric	Cumulative risk by age 75	7–8% [5]	8% [5]	5% [4]	0% [54]	*MLH1/MSH2* warrant attention
Prostate	Cumulative risk by age 75	17% [5]	32% [5,55]	18% [4]	0% [54]	Elevated risk in *MSH2*
Pancreas/Biliary	Cumulative risk by age 75	10% [5]	0.5–2% [5]	1% [4]	0% [54]	Marked predilection in *MLH1*

Table note: Data synthesized from the PLSD series (2018–2025) [4,5,53,54,55] and the surgical strategy analysis [59]. Risk ranges for MLH1/MSH2 reflect inter-study differences in estimates [5,53], which may partly arise from methodological variations including differences in ascertainment criteria, follow-up duration, and population composition across contributing centers; MSH6 data are primarily from Dominguez-Valentin et al. [4] and the PLSD mortality report [55]; PMS2 data are from Ten Broeke et al. [54]. Risk values are sex-combined or range values. Within-genotype heterogeneity driven by sex, family history, modifier genes, and surveillance adherence is not captured by these population-level estimates. Definitions of urinary tract cancers vary by study ([5] includes bladder cancer; [55] includes only upper tract).

**Table 7:** Impact of intervention strategies on clinical outcomes in LS (PLSD data).

Panel A: Surgical Strategy and Metachronous CRC Risk (Cumulative Risk by Age 75)					
Gene	Segmental Resection	Extended Resection		Absolute Risk Reduction	*p* Value
*MLH1*	69.1%	25.1%		44.0%	<0.05
*MSH2*	65.4%	14.7%		50.7%	<0.05
*MSH6*	31.9%	0%		31.9%	0.051
**Panel B: Colonoscopy surveillance intervals and cancer stage (overall comparison *p* = 0.34)**					
**Interval**	**Cases (*n*)**	**Stage I (%)**		**Stage II (%)**	**Stages III–IV (%)**
<1.5 years	36	61.1		22.2	16.7
1.5–2.5 years	93	49.5		31.2	19.4
2.5–3.5 years	56	53.6		37.5	8.9
>3.5 years	33	36.4		48.5	15.1
**Panel C: Cancer-related mortality (cumulative by age 75)**					
**Cancer type**	***MLH1***	***MSH2***	***MSH6***	***PMS2***	**% of total LS deaths**
Colorectal	6–8%	5–9%	2–3%	1–2%	36%
Non-colorectal	—	—	—	—	64%

Table note: Panel A from the PLSD 2025 surgical strategy analysis [59], including 8438 carriers; Panel B from Seppälä et al. (2019) on interval–stage associations [61], including 218 CRC cases; Panel C from Dominguez-Valentin et al. (2023) mortality analysis [55], including 8500 carriers and 71,713 person-years of follow-up.

**Table 8:** Immunotherapy-relevant molecular features and their clinical implications.

Panel A: Immunogenicity Determinants and Immune Microenvironment Features Relevant to ICIs in LS						
Feature	*MLH1*	*MSH2*	*MSH6*	*PMS2*	Refs.	Clinical Implication
TMB (mut/Mb)	~25	~47	NR	NR	[49]	Higher TMB correlates with ICI response
Indel burden	High (theoretical)	High (theoretical)	Low	Intermediate	[32,42,49]	Indel generate immunogenic FSDNs
FSDN load	High (theoretical)	High (theoretical)	Low	Intermediate	[32,42]	Primary target for neoantigen vaccines
SNV-derived neoantigens	Moderate	Moderate	High	Moderate	[42]	May partially compensate for low FSDNs in MSH6
CD8+ TIL density	NR	NR	NR	Lower	[52]	Predicts ICI response
**Panel B: Immune escape mechanisms, hypothesized genotype-associated ICI sensitivity, and known resistance factors in LS**						
**Feature**	***MLH1***	***MSH2***	***MSH6***	***PMS2***	**Refs.**	**Clinical Implication**
B2M loss	~28% (LS overall, not stratified by gene)				[89]	Mediates acquired ICI resistance
Hypothesized sensitivity	High	Highest	Potentially reduced	Intermediate	[49]	Requires prospective validation
Supporting rationale	High TMB/NAL; active immune infiltration	Highest TMB/NAL	Low Indel → fewer FSDNs	Lower immune infiltration	—	See Section 4.4.2
BRAF V600E (sporadic MLH1)	Common in sporadic MLH1-methylated CRC				[83]	mOS 19 vs. 113 months
Mucinous histology	Associated Low ORR				[60]	Consider combination therapy

Table notes: TMB, tumor mutational burden; Indel, insertion/deletion; FSDNs, frameshift-derived neopeptides; SNV, single-nucleotide variant; TIL, tumor-infiltrating lymphocyte; ICI, immune checkpoint inhibitor; NAL, neoantigen load; ORR, objective response rate; mOS, median overall survival; dMMR, mismatch repair deficient; MSI-H, microsatellite instability-high; NR, not reported. Critical caveat: “Hypothesized ICI response” is inferred from molecular features. No prospective trials have directly compared ICI efficacy across MMR genotypes in LS. Clinical decisions should be based on dMMR/MSI-H status, not genotype alone. *MSH6* and *PMS2* TMB values have not been separately reported in the literature; reference [49] reports data by heterodimer pairs only.

## Data Availability

No new data were created or analyzed in this study. Data sharing is not applicable to this article.

## Abbreviations

The following abbreviations are used in this manuscript:

ACMG/AMP	American College of Medical Genetics and Genomics/Association for Molecular Pathology
C-index	Concordance index
CNS	Central nervous system
CNV	Copy number variation
CRC	Colorectal cancer
c-miRs	Circulating microRNAs
cn-LOH	Copy number neutral loss of heterozygosity
dMMR	Defective MMR
EC	Endometrial cancer
EHR	Electronic health record
EHTG/ESCP	European Hereditary Tumour Group/European Society of Coloproctology
ESMO	European Society for Medical Oncology
FSDNs	Frameshift-derived neopeptides
GBM	Glioblastoma
HLA	Integrate human leukocyte antigen
ICI	Immune checkpoint inhibitors
Indel	Insertion/deletion
InSiGHT	International Society for Gastrointestinal Hereditary Tumours
IHC	Immunohistochemistry
LS	Lynch syndrome
LLS	“Lynch-like” syndrome
MAIT	Mucosal-associated invariant T
MDT	Multidisciplinary team
MLPA	Multiplex ligation-dependent probe amplification
MMR	Mismatch repair
mOS	Median overall survival
MSI	Microsatellite instability
MSS	Microsatellite stable
Mut/Mb	Mutations per megabase
NAL	Neoantigen load
NCCN	National Comprehensive Cancer Network
NMD	Nonsense-mediated decay
NGS	Next-generation sequencing
NR	Not reported
NPV	Negative predictive value
ORR	Objective response rate
OS	Overall survival
PD-L1	Programmed death-ligand 1
PFS	Progression-free survival
PLSD	Prospective Lynch Syndrome Database
pCR	Pathological complete response
PGS	Polygenic risk scores
pMMR	Proficient MMR
SIR	Standardized incidence ratio
SNV	Single-nucleotide variants
SSLs	Sessile serrated lesions
TMB	Tumor mutation burden
TIL	Tumor-infiltrating lymphocyte
TME	Tumor microenvironment
UC	Urothelial carcinoma
UPS	Undifferentiated pleomorphic sarcoma
UTUC	Upper tract urothelial carcinoma
VUSs	Variants of uncertain significance
VOCs	Volatile organic compounds
WES	Whole-exome sequencing
